# A Conservative Approach to Clinical Management of Syringomyelia

**DOI:** 10.7759/cureus.96661

**Published:** 2025-11-12

**Authors:** Kelli M Schindel, Sabrina Bustos, Susana Villate Prieto

**Affiliations:** 1 Family Medicine, Liberty University College of Osteopathic Medicine, Lynchburg, USA; 2 Family Medicine, Bon Secours Mercy Health, Norfolk, USA

**Keywords:** atypical back pain, chronic pain management, conservative medical management, preferred imaging modality, syringomyelia

## Abstract

Syringomyelia is an abnormal collection of cerebrospinal fluid in the spinal cord. This fluid-filled cavity can elongate or expand over time, causing paresthesia, paralysis, or pain. We review the case of a 68-year-old woman with a thoracic syrinx from T5 to T11. The patient has a long-standing history of cervicalgia, lumbar radiculopathy, nerve root impingement, and degenerative disc disease. After years of chronic pain, the patient presented with worsening right-sided back pain that radiated to the umbilicus, which was initially treated with conservative pharmacotherapy. Due to inadequate pain management, etiologies outside of the previously diagnosed radiculopathy were considered. An incidental finding on an MRI of the thoracic spine demonstrated a thoracic syrinx extending from T5 to T11. A standardized approach to conservative clinical management for syringomyelia has not been established, which has led to difficulty in patient care for individuals suffering from chronic, symptomatic syrinx spinal cord disorders.

## Introduction

Syringomyelia occurs in the presence of Chiari malformation type I, a condition in which the hindbrain extends into the spinal column, creating a syrinx [1,2]. This fluid-filled cavity demonstrates a focal dilation of either the central canal or the spinal parenchyma. Although rare, a syrinx may form in the absence of Chiari malformations as a result of infection, inflammation, neoplasia, or trauma [3]. In these instances, syringomyelia is characterized by abnormal cerebrospinal fluid accumulation leading to the formation of a syrinx. The first presenting sign of this disorder is usually sensory symptoms such as pain and temperature discrepancies; however, the size, location, and extent of the fluid-filled cavity determine symptom severity and nature [4,5]. 

Syringomyelia formation can be further classified into three distinct groups: communicating, non-communicating, and extracanalicular. Due to our patient's history of trauma to her spine, the pathophysiology of her lesion is likely extracanalicular, which typically presents in the watershed areas of the spinal cord: T1 to T3 and T6 to T9. Extracanalicular syrinx cavities are often associated with myelomalacia, a softening of the spinal cord occurring after hypoperfusion, myelopathy, or acute injury, as in this patient [6,7].

The prevalence of Chiari malformation type I ranges from three to eight per 100,000, with syringomyelia presenting in 65% of these cases [8]. However, our patient’s case of isolated syringomyelia is exceedingly rare considering syringomyelia without Chiari malformation type I has a prevalence of 8.4 per 100,000 to 0.9 per 10,000 [9]. Due to the availability of MRI, there has been an increase in the number of incidental findings of syringomyelia. It has also caused an increase in the number of asymptomatic cases of syringomyelia, which is currently 22.7% of cases [10]. Increased MR quality allows imaging to reflect syrinx cavities before typical symptoms present. The rarity of these cases has led to a gap in existing guidance on how to manage patients with isolated syringomyelia.

## Case presentation

A 68-year-old woman with a history of cervicalgia, chronic lumbar back pain with nerve impingement at C6 to C7 and L4 to L5, and degenerative disc disease at T11 to T12 presented to the clinic to establish care. In addition, she had a history of chronic back pain due to a traumatic event that caused an injury to her spine in 2004. Pain management for the past year included acetaminophen and ibuprofen. Further recommendations for pain control included baclofen and diclofenac. Her chronic back pain continued despite these medication changes, leading to an accidental overdose from baclofen ingestion. 

A year later, the patient presented to the emergency room for right lower quadrant abdominal pain that radiated to her lower back. After ruling out acute, life-threatening abdominal pathology, she was discharged on prednisone, acetaminophen, and cyclobenzaprine for her back pain. She returned to the emergency room the following day because of continued lower back pain. She was prescribed lidocaine patches, ibuprofen, acetaminophen, and oxycodone and discharged. 

**Figure 1:**
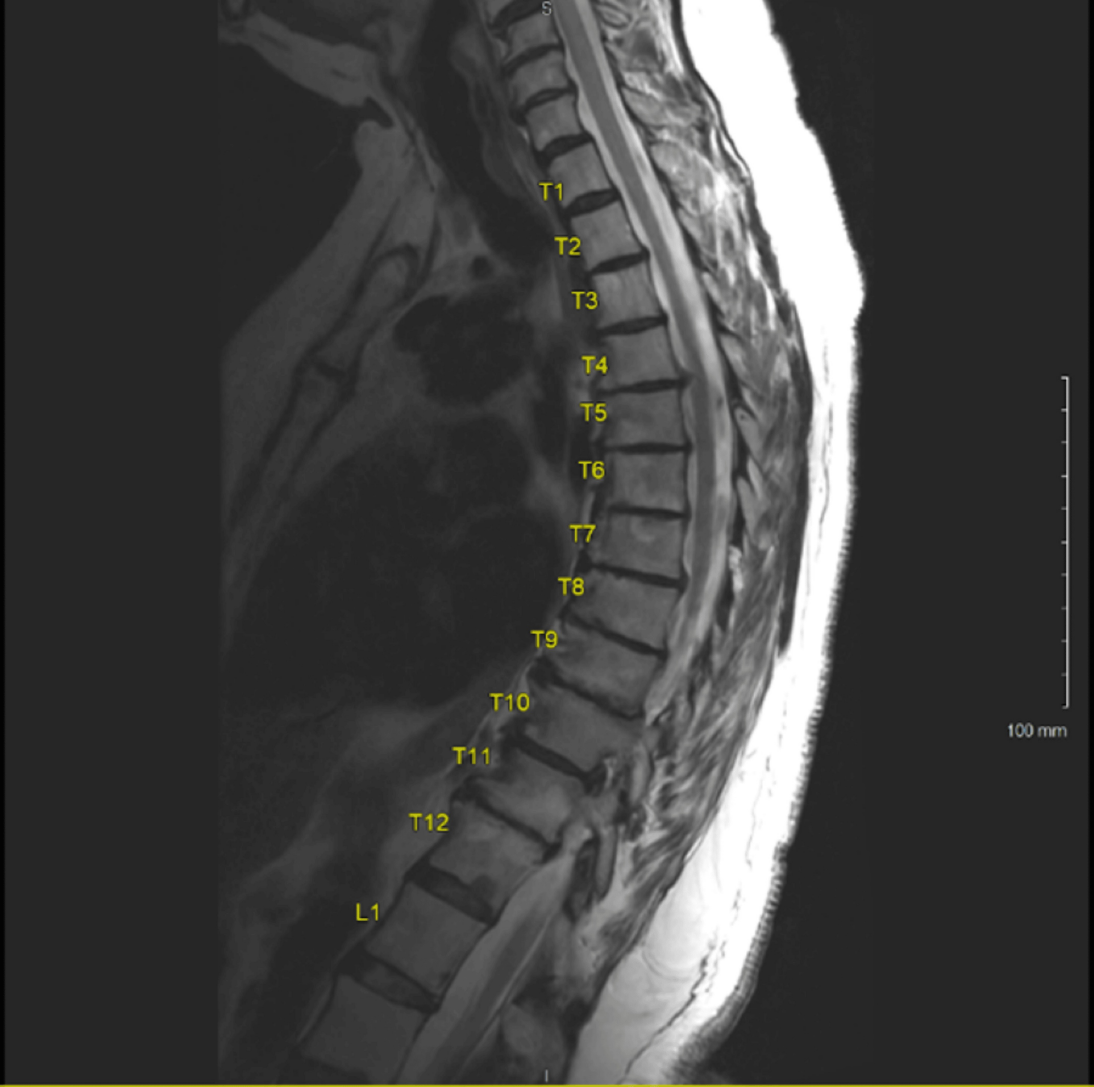
MRI of the thoracic spine with a spinal cord thoracic syrinx spanning T5 to T11

The patient presented to the clinic for a follow-up appointment after her visits to the emergency room and complained of continued right-sided back pain that radiated around her side towards the lateral aspect of the umbilicus. Pain was provoked by tensing the abdominal wall and changes in position. Slight relief was achieved with pain medication and a heating pad. At her one-week follow-up appointment, the patient continued to have significant lower back pain that radiated to her abdomen. Suspicion of muscle spasms in her back prompted the prescription of baclofen, diazepam, and ketorolac. At her one-week follow-up, she continued to have significant pain. She was given a provisional diagnosis of anterior cutaneous nerve entrapment based on her presenting symptoms. Gabapentin was prescribed for symptoms of thoracic radiculopathy, and an MRI was ordered. At the two-month follow-up, she reported improvement of back pain radiating to the abdomen; however, an MRI of the thoracic spine included an incidental finding of a thoracic cord syrinx spanning the T5 to T11 vertebral body levels (Figure 1). The patient was advised to continue gabapentin for pain management and was given a referral to neurosurgery.

## Discussion

The diagnostic evaluation of syringomyelia varies based on the clinical presentation, making it difficult to identify. Although it is typical for acquired syringomyelia to be asymptomatic, most symptomatic patients present with bilateral motor and sensory deficits that prompt diagnostic imaging such as MRI, 3D constructive interference in steady state (CISS) sequences, or four-dimensional (4D) phase contrast (PC) MRI. If a syrinx is found, imaging to rule out life-threatening etiologies must include MRI of the lumbosacral region and the craniocervical junction. In addition, the use of contrast allows physicians to identify intramedullary and extramedullary lesions that may determine the course of treatment. 

**Figure 2:**
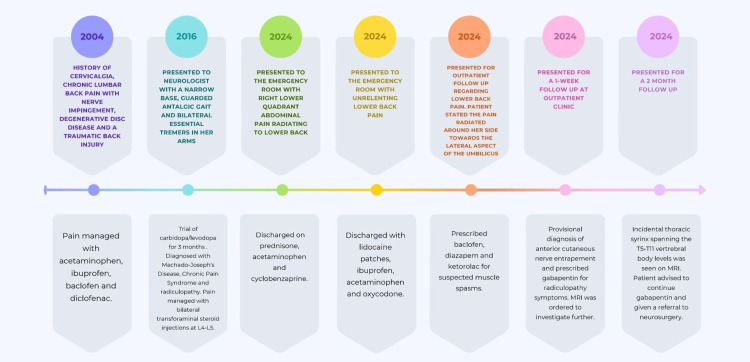
Timeline of events, diagnoses and therapeutic management

Due to the nature of our patient's chronic back pain, the diagnostic evaluation was broad. Years prior to establishing care at our clinic, she presented to her neurologist with a narrow base and a guarded and antalgic gait, with bilateral essential tremors in her arms, prompting a diagnosis of idiopathic Parkinson's disease. Her back pain seemed to be associated with the traumatic event from 2004. An MRI of the C-spine, L-spine, and brain showed very minimal disk protrusion at C5-C6 and C6-C7, minimal disk protrusion at L4-L5 and L5-S1, and no spinal cord abnormality. In addition, the brain MRI showed no abnormalities except for subtle signs of loss of hyperpigmentation in the pars reticulata of the left substantia nigra. The locus coeruleus and pons were normal, and there was no white matter disease in the posterior fossa to suggest olivopontocerebellar atrophy. A trial of carbidopa/levodopa for three months provided minimal relief. Upon return, the review of systems was significant for tremors, stiffness, and slowness, as well as back pain, despite pharmacologic treatment. A negative brain MRI and a trial of targeted pharmacotherapy made Parkinson’s disease less likely. A diverse overview of her symptoms led to the differential diagnoses of Machado-Joseph disease, chronic pain syndrome, and radiculopathy. Maintenance of the patient's symptoms included bilateral transforaminal steroid injections at L4-L5 and analgesics. The patient’s new symptoms of right flank pain that radiated to the lateral umbilicus and muscle spasms resembled anterior cutaneous nerve entrapment. This prompted further imaging of the thoracic spine and abdomen (Figure 2). 

Current evidence lacks a clear understanding of how to manage patients with isolated syringomyelia. However, in every case, providers are faced with the decision of whether surgical or conservative management is appropriate. Surgical procedures should be considered for those with large, symptomatic syrinx cavities; those with an underlying cause (such as a Chiari malformation); or posttraumatic syringomyelia with motor deficits [10]. Neurosurgical management offers two types of interventions: emptying the syrinx into other cavities/tubes or locating the area of obstruction and restoring cerebrospinal fluid flow [3]. Although patients with symptomatic syringomyelia may opt for surgery, complications include acute hydrocephalus, bacterial meningitis, cerebrospinal fluid leak, and chemical meningitis. Pain and somatic sensory deficits can also persist after surgical treatment [10].

Conservative treatment focuses on managing syringomyelia-related symptoms that the individual experiences. Although active conservative interventions can potentially lead to improvement in activities of daily living and quality of life, supporting research is limited [11]. Behavioral changes such as avoiding straining, flexing the neck, and breath-holding spells can decrease venous pressure, therefore alleviating symptoms [12]. Many symptomatic individuals, such as our patient, require pain control for management of this disorder. Conventional pain medication has been shown to be ineffective in managing neuropathic pain. Studies show that tricyclic antidepressants or antiepileptics could be more effective options for analgesic treatment [13]. Physical therapy has also been found to help manage pain and improve quality of life [9]. Regardless, patients will need a multidisciplinary approach that involves their primary care providers, neurosurgeons, and pain specialists to effectively manage their condition.

## Conclusions

The rarity of isolated syringomyelia has led to a gap in the scientific literature in terms of how to manage this patient population through conservative measures. With the number of incidental findings increasing, the need for how to treat these individuals rises as well. Asymptomatic patients and patients with atypical symptoms, such as chronic pain, as in our current patient, are being recognized more. Typically, the revelation of an asymptomatic syrinx through routine MRI investigation is considered a variant of normal anatomy; however, in any event, syringomyelia has the potential to transition from asymptomatic to symptomatic at any point. Due to this, the support and management for patients with syringomyelia needs to be established so physicians can provide standardized care. Specifics on effective physical therapy techniques and appropriate analgesic therapy should be further explored to help manage syringomyelia-related symptoms to improve quality of life.
